# Clinical and histological characterization of 19 chondro-osseous respiratory epithelial adenomatoid hamartomas and 2 respiratory epithelial adenomatoid hamartomas in dogs

**DOI:** 10.1177/03009858251346221

**Published:** 2025-06-26

**Authors:** Ashley Forster, David Holt, Amy C. Durham

**Affiliations:** University of Pennsylvania, Philadelphia, PA

**Keywords:** canine, chondro-osseous respiratory epithelial adenomatoid hamartoma, dog, nasal, polyp, respiratory epithelial adenomatoid hamartoma, sinus

## Abstract

Nasal biopsies from 21 dogs diagnosed with chondro-osseous respiratory epithelial adenomatoid hamartomas or respiratory epithelial adenomatoid hamartomas were reviewed. Associated lesions included angiomatous tissue (4/21), seromucinous gland proliferation (2/21), and polyps (3/21), and all had chronic inflammation. Dogs had epistaxis (14/21), sneezing (9/21), decreased airflow (8/21), congestion (6/21), and discharge (5/21). In addition to a mass lesion, computed tomography findings (*n* = 19) included turbinate lysis (10/19), cribriform plate and orbit erosion (4/19), and contralateral extension (8/19). In 16 dogs with outcome data collected 0–49 months after diagnosis, 13 had continued respiratory symptoms, 11 of which received medical management; 2 of the 3 dogs with improvement had radiotherapy/radiofrequency procedures after biopsy. Eight dogs were alive, 5 were euthanized due to the hamartoma (median survival 9.3 months), 2 died from unknown causes, and 1 died from a seizure. Nasal hamartomas are often locally destructive mass lesions that cause recurrent upper respiratory symptoms and may require more aggressive therapeutic interventions for disease control. Diagnosing nasal hamartomas requires integrating the clinical history, imaging results, adequate biopsy sampling of the mass, and the presence of characteristic histologic features.

A hamartoma is a non-neoplastic, mass-like lesion composed of disorganized tissue indigenous to the site in which it grows. Canine nasal hamartomas are uncommonly reported, space-occupying, soft-tissue lesions in the nasal cavity. Descriptive cases of chondro-osseous respiratory epithelial adenomatoid hamartomas (COREAHs) in the veterinary literature include a case series of 3 dogs, 2, 8, and 10 years of age, and a single case report in a 5-year-old dog.^1,6^ There are two single-case reports of respiratory epithelial adenomatoid hamartomas (REAHs) in a 6-month-old and a 4-year-old dog.^7,9^ The REAHs were described as polypoid masses comprised of respiratory epithelial-lined, edematous, fibrovascular tissue with variably distended glandular or adenomatoid structures lined by similar epithelium. The COREAHs were described as having similar gross and histological features but were organized around chondro-osseous cores. The dogs had signs of exophthalmia, epistaxis, and difficulty breathing due to the presence of the nasal mass, and imaging showed evidence of regional bony lysis of turbinates and surrounding skull bones. Metastasis has not been reported in any dogs.

In addition to REAHs and COREAHS, the hamartoma subtypes described in humans include seromucinous hamartoma (SH) and nasal chondromesenchymal hamartoma (NCH).^4,8^ SHs are characterized by a proliferation of haphazardly arranged seromucinous glands and may have areas resembling REAH. NCH consists of a mesenchymal proliferation of spindle-shaped stromal cells amid islands of mature cartilage with osteoid formation. REAH, SH, and COREAH occur in adult patients, whereas NCH is associated with a mutation of the tumor suppressor gene *DICER1* and is mainly reported in infants.^3,8^ The key histologic features of these hamartomas, as described in dogs and humans, are summarized in Supplemental Table S1.

Despite the limited number of nasal hamartoma cases reported in dogs, these entities have been recognized in diagnostic laboratories since their initial descriptions were published. This study aimed to describe the clinical and histological characteristics and clinical outcomes of canine nasal hamartomas in a larger cohort of cases.

Biopsy archives from the Penn Vet Diagnostic Laboratory were searched from 2016 to 2023 for all canine nasal biopsy reports that included the word “hamartoma” in the diagnosis or comment. On the submission forms, biopsy samples were denoted as being obtained via rhinotomy (excisional) or cup forceps, either via rhinoscopy or transnasal approaches (incisional). The hematoxylin and eosin–stained slides of the cases that met the initial inclusion criteria were reviewed by a single board-certified pathologist (ACD) for histologic features of nasal hamartomas reported in previous canine case studies.^1,6,7,9^ A second round of slide and submission form review (AF, ACD) applied more stringent criteria to exclude cases in which sufficient tissue was not available for a definitive diagnosis, had uncertain sampling of the mass lesion during biopsy retrieval, or had additional lesions concerning for carcinoma or other neoplasia. In addition to using histologic criteria reported in dogs,^1,6,7,9^ slides were evaluated based on the classification used in the human literature (Supplemental Table S1).^4,8^ At this time, slides from five cases that had either previous (*n* = 3) or subsequent (*n* = 2) nasal biopsies performed were also evaluated. Given there is histologic overlap between nasal hamartomas and non-hamartomatous reactive processes, in addition to the histologic criteria, all cases required a clinical history compatible with a nasal lesion; identification of a nasal mass grossly, surgically, and/or via computed tomography (CT) imaging; and appropriate sampling of the mass lesion. After the review process, 21 cases were identified for inclusion in the study.

Clinical data from each case were mined from the biopsy submission forms and available medical records, or were obtained via an emailed questionnaire or telephone interview with referring veterinarians, and included the following information: clinical signs, date of onset, progression, treatment type, occurrence and duration of clinical remission, and whether the patient was still alive at the time of the survey or the date and reason for euthanasia/death.

Patients included 7 spayed females, 13 castrated males, and 1 intact male across several breeds: 4 mixed breed dogs, 3 Labrador Retrievers, and 1 each of American Pit Bull Terrier, Beagle, Boxer, Pembroke Welsh Corgi, Standard Poodle, Collie, Yorkshire Terrier, Husky, German Shepherd Dog, Great Pyrenees, Catahoula Leopard Dog, Weimaraner, English Springer spaniel, and Australian Cattle Dog. Dogs’ ages ranged from 3 to 16 years, with a median and mean of 8 years.

Clinical signs in affected dogs included epistaxis (14/21, 66%), sneezing (9/21, 43%), decreased or absent airflow from the nostril(s) (8/21, 38%), stertor (7/21, 33%), nasal congestion (6/21, 29%), serous nasal discharge (5/21, 24%), increased respiratory effort (3/21, 14%), ocular discharge (3/21, 17%), facial swelling (3/21, 17%), and ocular swelling (1/21, 5%). Nineteen patients had CT imaging performed (Fig. 2a), which, in addition to the mass lesion, showed turbinate lysis (10/19, 53%), extension into the contralateral nasal passage (8/19, 42%), lysis of the cribriform plate (4/19, 21%), erosion of the wall of the orbit (4/19, 21%), extension into the nasopharynx (4/19, 21%), and deviation of the nasal septum (1/19, 5%). The masses were predominantly unilateral, with 11 originating on the right side, 9 originating on the left side, and 1 in which laterality was not reported. Biopsy specimen types listed on the submission forms included rhinotomy (*n* = 2) and cup forceps (*n* = 18), and one patient expelled the nasal mass.

Histologically, most cases were diagnosed as COREAHs (*n* = 19), and 2 dogs were diagnosed with REAHs. COREAHs were characterized as masses lined by pseudostratified, ciliated respiratory epithelium with stromal edema, adenomatoid or gland-like structures lined by respiratory epithelium that likely represented invaginations of the surface epithelium, and disorganized islands of a mixed chondro-osseous matrix (Supplemental Table S1, Figs. 1a–c). The adenomatoid structures were variably ectatic and filled with eosinophilic proteinaceous to lightly basophilic mucinous material with neutrophils and cell debris (Fig. 1a). The chondro-osseous matrix was composed of disorganized bone trabeculae with irregularly spaced osteocytes in lacunae, multifocally lined by variably prominent osteoblasts, as well as variably shaped hyaline cartilage foci consisting of a blue-gray to amphophilic chondroid matrix with embedded individualized or clustered chondrocytes (Figs. 1b, c). The distribution, amount, and proportion of osseous and chondroid tissue were variable in cases of COREAH. There were mixed degrees of lymphoplasmacytic inflammatory cell infiltration.

**Figure 1.**
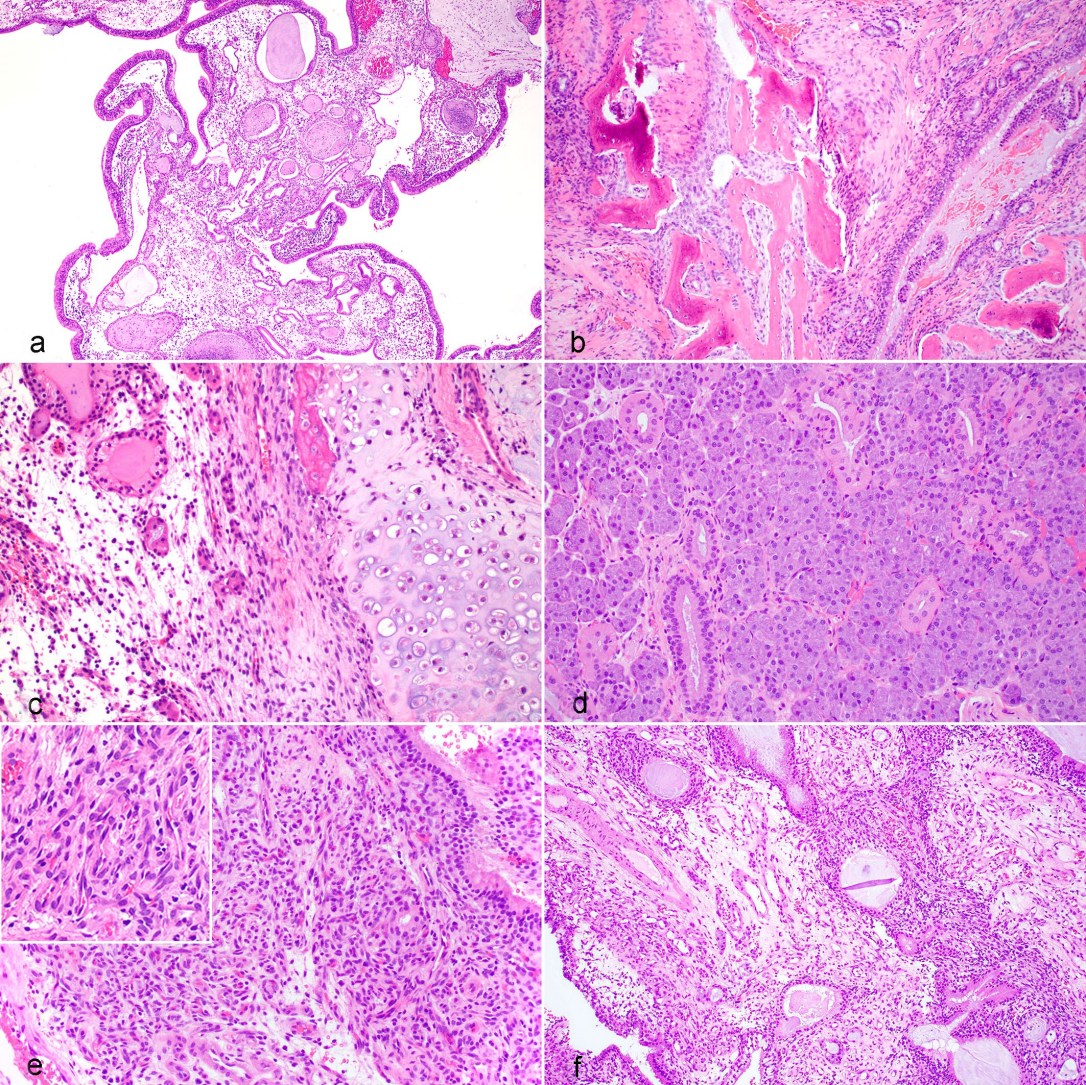
Histopathology of nasal hamartomas in dogs. Hematoxylin and eosin. (a) Polypoid mass lined by respiratory epithelium with stromal edema and distended adenomatoid/gland-like structures. (b) Osseous core within a chondro-osseous respiratory epithelial adenomatoid hamartoma (COREAH). (c) Higher magnification of chondro-osseous matrix within a COREAH. (d) Marked seromucinous gland hyperplasia in a COREAH. (e) Angiomatous tissue in a COREAH. Inset: higher magnification of angiomatous tissue. (f) Respiratory epithelial adenomatoid hamartoma (REAH) with stromal edema and distended adenomatoid/gland-like structures; the chondro-osseous matrix seen in COREAH is absent.

Additional characteristics were noted in a subset of the 19 COREAHs. Cases 9 and 14 had foci resembling SH, as described in the human literature (Fig. 1d).^4^ In addition to the features of COREAHs, these lesions had multifocal expansions of seromucinous glands containing variable amounts of eosinophilic secretory material. Cases 6, 13, 15, and 21 had confluent foci of dense angiomatous tissue consisting of tightly packed blood vessels lined by hypertrophied endothelium (Fig. 1e). The two REAHs were masses lined by respiratory epithelium with stromal edema and similar distended adenomatoid structures, but without the chondro-osseous foci in the sampled tissues (Fig. 1f). Criteria of malignancy, such as nuclear or cellular atypia and mitotic activity, were not observed in any population.

Data on treatments following the biopsy procedures were available for 16 of 21 dogs (76%); 3 dogs (19%) did not undergo additional treatment following surgery. In the remaining 13 dogs, medical treatments included antibiotics (6/13, 46%), nonsteroidal anti-inflammatory drugs (4/13, 31%), corticosteroids (3/13, 23%), and trimeprazine with prednisolone (Temaril-P, Zoetis) (2/13, 15%). Additional interventions included sinusotomy (2/13, 15%), CyberKnife radiation therapy (1/13, 8%), an ablation procedure (1/13, 8%), and surgical debulking (1/13, 8%).

Five cases had either previous (*n* = 3) or subsequent (*n* = 2) biopsies performed from the same nostril that was diagnosed with the nasal hamartoma (Table 1). Case 2 had a previous biopsy of a nasal polyp approximately 1 year before the COREAH diagnosis. The nasal polyp had the characteristic respiratory epithelium overlying a markedly edematous submucosal stroma with mucin accumulation, lymphangiectasia, and mild mixed inflammation (Supplemental Table S1).^11^ Case 3 had 3 previous biopsies, ranging from 5 to 17 months before the COREAH diagnosis, which were diagnosed as rhinitis and nasal polyps. Case 9 had a biopsy 4 months before the original COREAH diagnosis that showed severe chronic suppurative rhinosinusitis.

**Table 1.** Previous and subsequent biopsy results from dogs diagnosed with chondro-osseous respiratory epithelial adenomatoid hamartoma (COREAH).

Case	Time of Biopsy Relative to Sinonasal Hamartoma Diagnosis	Biopsy Result
2	13 months prior to COREAH diagnosis	Nasal polyp
3	17 months prior to COREAH diagnosis	Nasal polyp
3	11 months prior to COREAH diagnosis	Severe chronic-active rhinitis with bone remodeling, edema, and glandular ectasia
3	5 months prior to COREAH diagnosis	Severe chronic-active rhinitis with edema, glandular ectasia, and rupture
5	22 months following COREAH diagnosis	Chronic rhinitis with irregular fibroplasia and bone modeling
9	4 months prior to COREAH diagnosis	Severe chronic suppurative rhinosinusitis
14	1 month following COREAH diagnosis	Nasal polyp with hemorrhage, fibrosis, and edema

Two cases had biopsies following the initial histologic diagnosis. Case 5 had a cup forceps biopsy 2 years following the original COREAH diagnosis, also made via cup forceps, due to continued nasal clinical signs but no mass. This sample was diagnosed as chronic rhinitis with irregular fibroplasia and bone modeling; bone modeling was characterized as thinned, mature turbinate bone with basophilic reversal lines lined by plump osteoblasts and rare osteoclasts. These tissues were small, 2- to 3-mm fragments that lacked the characteristic features of COREAH (distended gland-like structures lined by respiratory epithelium and chondro-osseous cores) and, in conjunction with the lack of a clinically identifiable mass, were interpreted as reactive tissue based on the samples examined, likely related to the previous surgical intervention. Case 14 had a rhinoscopic biopsy 1 month following the COREAH diagnosis made via rhinotomy that showed the characteristic lesions of nasal polyps with hemorrhage, fibrosis, and edema.^11^ This patient’s CT scan from the original COREAH is seen in Fig. 2a, and the polyps from the subsequent biopsy were visualized on rhinoscopy (Fig. 2b).

**Figure 2.**
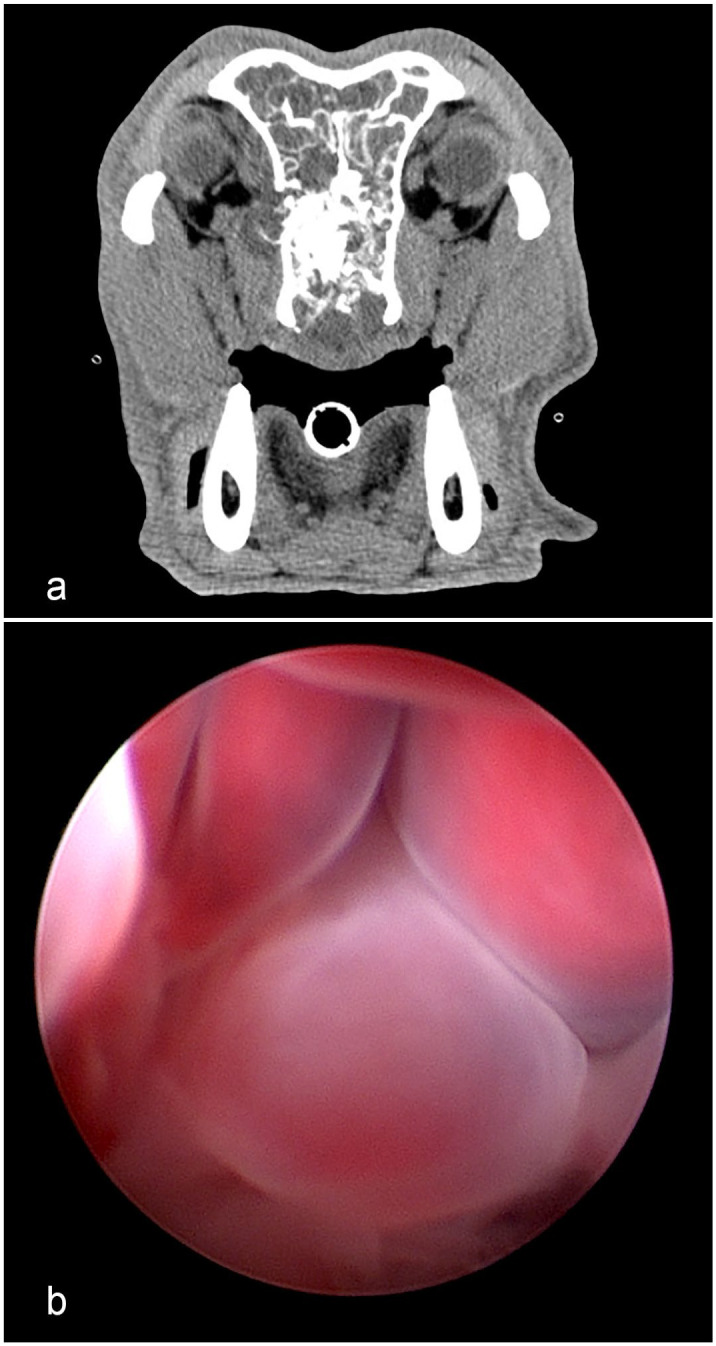
Clinical imaging of nasal hamartoma. (a) Computed tomographic image from a dog diagnosed with a chondro-osseous respiratory epithelial adenomatoid hamartoma (COREAH) reveals a soft-tissue mass that fills the right nasal cavity with deviation of the nasal septum and extension into the left nasal passage. There is loss of a large portion of the nasal turbinates and lysis of the right nasal bones. (b) Repeat rhinoscopy performed 1 month after the initial biopsy. Intraprocedural images show multiple nasal polyps.

Follow-up information via surveys on patient outcomes was available for 16 of 21 cases (76%). The time between the biopsy diagnosis and receipt of the follow-up information ranged from 0 to 49 months (mean 12 months). Thirteen dogs (81%) had no improvement of their respiratory symptoms after diagnosis. Eleven of these dogs received medical treatment after diagnosis, and their respiratory symptoms persisted. Two of these 11 cases (cases 9 and 14) were diagnosed via rhinotomy. Of the three cases that did not have reported persistence of respiratory symptoms at 5–6 months after biopsy, 2 had more aggressive treatments, including sinusotomy and ablation procedure (case 17) and CyberKnife procedure (case 16), while in the third, only a 15 × 7-mm biopsy was removed via cup forceps (case 18).

At the time of the survey, 8 dogs were alive (50%), and 8 were deceased (50%). Of the 8 deceased dogs, 5 were euthanized 0–21 months after the biopsy due to decreasing quality of life related to respiratory signs from the nasal mass; 2 dogs died from an unknown cause; and 1 died from a seizure that was not suspected to be related to the COREAH.^1^ The overall median survival time for the 8 deceased dogs was 9.3 months; the median survival time for the 7 dogs euthanized specifically due to the nasal symptoms was 6.8 months. A complete dataset, including age, presenting clinical signs, CT results, biopsy sample type/size, diagnosis, postbiopsy treatment, clinical signs following diagnosis and treatment, and survival, is presented in Supplemental Table S2.

The etiology of nasal hamartomas in dogs is unclear. In humans, sinonasal hamartomas are considered benign reactive lesions by the WHO Classification of Head and Neck Tumors.^8^ REAHs are the best described and most common in people, occur in adults with a male predisposition, and have been associated with inflammatory processes such as rhinosinusitis and polyps.^8,12^ It is reported that 50.1% of REAHs had concurrent nasal polyposis.^10^ The three dogs in which biopsies were taken before the COREAH diagnoses had chronic rhinitis/rhinosinusitis, and/or nasal polyps. The nasal polyp diagnoses, both in prior and subsequent biopsies, are of particular interest as a previous study has shown that 16% of dogs with nasal polyps had underlying neoplasia.^11^ In the current study, the evidence of previous or subsequent nasal polyps suggests a possible association, including the possibility that hamartomas may obstruct lymphatic drainage and lead to marked edema and polyp formation. There are no reports in the human or veterinary literature that directly implicate surgical biopsy as a predisposing factor for hamartoma formation. Strong evidence for a congenital or hereditary cause for the majority of sinonasal hamartomas in humans is not available, except for NCH, which is associated with a *DICER1* mutation and is diagnosed most often in infants.^3,8^ The population of dogs in this study included adults with a mean age of 8 years; thus, congenital causes may be less likely.

The 4 dogs with co-occurring angiomatous tissue had some histological features similar to a nasopharyngeal angiofibroma, which is a benign but locally invasive tumor that has been reported in a series of 13 dogs^2^ and 1 case report.^5^ Notably, one case report of a COREAH in a 4-year-old French bulldog had an initial tentative biopsy diagnosis of angiofibroma.^9^ Two dogs had increased seromucinous gland tissue, somewhat resembling SHs in humans, which are noted to co-occur with REAHs. It is unclear if the angiomatous tissue and seromucinous gland proliferation represent distinct lesions like angiofibroma and SH. However, the authors speculate that these features may be reactive processes related to local tissue disturbances and, thus, part of a spectrum of lesions present within canine nasal hamartomas.

CT imaging showed that nasal hamartomas in dogs are often destructive lesions. This finding was consistent with the canine cases of COREAHs and REAH that were previously reported. This extent of bony lysis is not typical in human COREAH cases, and reports in humans indicate that REAHs and COREAHs rarely recur after surgical excision.

In this study, the upper respiratory clinical signs in the majority of dogs (13/16, 81%) persisted, even in the 2 dogs that had excisional biopsies (rhinotomies) performed. Of note, the 2 dogs with more aggressive postbiopsy treatments (e.g., sinusotomy/ablation procedure and Cyberknife therapy) did not have a recurrence of clinical signs at 5–6 months after procedures. Recent case reports described the resolution of clinical signs following a lateral rhinotomy and coblation, a process in which radiofrequency energy is applied, of a REAH in a 4-year-old French bulldog.^9^ Improvement but not resolution of clinical signs was reported following a dorsal rhinotomy with turbinectomy and debridement of a COREAH in a 5-year-old Yorkshire terrier.^1^ These data suggest that more aggressive therapeutic interventions may be the best option for the relief of persistent clinical signs in dogs with nasal hamartomas.

Nasal cavity masses in dogs include malignant neoplasms, such as adenocarcinoma, chondrosarcoma, and osteosarcoma, as well as more benign but clinically significant mass-like lesions, including nasal polyps, angiofibroma, and hamartomas. Nasal hamartomas are an important differential diagnosis for dogs with nasal diseases, especially those with locally destructive and recurrent nasal masses visualized on CT scans. Biopsy sampling of the mass with adequate tissue is crucial to assist in making a definitive diagnosis, as differentiating hamartomas from normal nasal tissue, nasal polyps, or non-hamartomatous reactive nasal lesions is challenging in small tissue fragments. Non-hamartomatous reactive or inflammatory lesions typically maintain nasal turbinate scroll-like architecture, and there may be submucosal edema and congestion; mixed inflammatory cell infiltrates; respiratory epithelial erosion, ulceration, or hyperplasia depending on the underlying process; seromucinous gland loss or hyperplasia; and thinned to fragmented turbinate bone with modeling changes (e.g., basophilic reversal lines, plump lining osteoblasts, and osteoclasts). Nasal hamartomas are distinguished as polypoid/bulbous masses that lack the delicate nasal turbinate structure and have the additional features of ectatic adenomatoid structures lined by respiratory epithelium and irregular variably sized chondro-osseous cores in cases of COREAH.

Given the histologic overlap between nasal hamartomas and other reactive nasal lesions, a diagnosis of hamartoma should not be given based solely on histopathology; it is critical to assimilate the clinical and histopathological data to avoid misdiagnosis or overdiagnosis. The diagnosis of hamartoma is dependent on the clinical findings of an expansile, polypoid, mass-forming, possibly destructive lesion within the nasal cavity, associated clinical signs, and a representative biopsy consisting of the characteristic histopathologic features of respiratory epithelial-lined edematous tissue with distended gland-like structures lined by the same epithelium, with (COREAH) or without (REAH) chondro-osseous cores.

There appears to be some variation in the biological behavior of nasal hamartomas. The appropriate treatment for these masses has yet to be determined; however, this limited case series suggests that dogs with more aggressive interventions may achieve complete resolution. Further investigations of the etiologies, associated and concurrent disease processes, and optimal treatment of nasal hamartomas in dogs are necessary.

## Supplemental Material

sj-pdf-1-vet-10.1177_03009858251346221 – Supplemental material for Clinical and histological characterization of 19 chondro-osseous respiratory epithelial adenomatoid hamartomas and 2 respiratory epithelial adenomatoid hamartomas in dogs Supplemental material, sj-pdf-1-vet-10.1177_03009858251346221 for Clinical and histological characterization of 19 chondro-osseous respiratory epithelial adenomatoid hamartomas and 2 respiratory epithelial adenomatoid hamartomas in dogs by Ashley Forster, David Holt and Amy C. Durham in Veterinary Pathology
