# Supplementary material for: Clinical and histological characterization of 19 chondro-osseous respiratory epithelial adenomatoid hamartomas and 2 respiratory epithelial adenomatoid hamartomas in dogs
Source: Vet Pathol. 2025 Jun 26;62(6):922–7. doi: 10.1177/03009858251346221 (PMC12534865; doi:10.1177/03009858251346221)
Supplement: sj-pdf-1-vet-10.1177_03009858251346221 – Supplemental material for Clinical and histological characterization of 19 chondro-osseous respiratory epithelial adenomatoid hamartomas and 2 respiratory epithelial adenomatoid hamartomas in dogs [file sj-pdf-1-vet-10.1177_03009858251346221.pdf]

Supplemental Materials

Clinical and histological characterization of 19 chondro-osseous respiratory epithelial adenomatoid hamartomas and 2 respiratory epithelial adenomatoid hamartomas in dogs

Ashley Forster, David Holt, Amy Durham

SUPPLEMENTAL TABLE S1. *Descriptive histological features of human and canine nasal non-malignant masses*

Entity	Histologic Characteristics
Chondro-osseous respiratory epithelial adenomatoid hamartoma (COREAH)	▪ Respiratory epithelial-lined polypoid mass of mature, disorganized nasal turbinate tissue<br>▪ Organized around cores of chondro-osseous matrix<br>▪ Distended adenomatoid/gland-like structures lined by respiratory epithelium (invaginations of surface epithelium)
Respiratory epithelial adenomatoid hamartoma (REAH)	▪ Respiratory epithelial-lined polypoid mass of mature, disorganized nasal turbinate tissue<br>▪ Lacks cores of chondro-osseous matrix<br>▪ Distended adenomatoid/gland-like structures lined by respiratory epithelium (invaginations of surface epithelium)
Seromucinous hamartoma (SH)	▪ Respiratory epithelial-lined polypoid mass<br>▪ Proliferation of haphazardly arranged seromucinous glands<br>▪ Variable presence of adenomatoid/gland-like structures lined by respiratory epithelium (invaginations of surface epithelium)
Nasal chondromesenchymal hamartoma (NCH)	▪ Respiratory epithelial-lined polypoid mass<br>▪ Haphazardly arranged spindle-shaped cells amid a collagenous stroma<br>▪ Islands of mature cartilage with focal osteoid formation
Angiofibroma	▪ Polypoid mass composed of vascular and fibrous stromal tissue<br>▪ Variably sized/ectatic, endothelial-lined blood vessels<br>▪ Collagenous stroma with fibroblasts<br>▪ Minimal atypia and mitotic figures
Nasal polyp	▪ Respiratory epithelial-lined polypoid mass<br>▪ Edematous submucosal stroma with mild mixed inflammatory infiltrate, mucin accumulation, and lymphangiectasia

SUPPLEMENTAL TABLE S2. *Age, clinical signs, CT results, histopathologic diagnoses, treatment, and outcomes.*

Case	Age (Yrs)	Clinical Signs	CT Results	Biopsy Type (largest tissue size[a])	Diagnosis	Treatment Post Biopsy	Persistent Respiratory Signs	Survival (months)
1	4	Stertor, sneezing; mass expelled during sneeze	N/A	Expelled (20x5 mm)	COREAH	LTFU	LTFU	LTFU
2	10	Serous nasal discharge, sneezing	Erosion of turbinates	Cup forceps (25x10 mm)	COREAH	LTFU	LTFU	LTFU
3	16	Absent nostril airflow, nasal discharge, epistaxis, sneezing, congestion, stertor, increased respiratory effort	N/A	Cup forceps (11x4 mm)	COREAH	Saline nebulization, piroxicam, debulking procedure	Persistent left-sided nasal discharge. Debulk temporarily relieved signs	Euthanized (12.7)
4	7	Epistaxis, sneezing, congestion, decreased nasal airflow	Lysis of turbinates, medial orbit, and nasal septum	Cup forceps (20x10 mm)	COREAH	LTFU	LTFU	LTFU
5	13	Coughing, gagging, throat clearing	Extension of mass into contralateral nasal cavity	Cup forceps (7x3 mm)	COREAH	Temaril-P	Persistent coughing	Alive (49)
6	13	Nasal congestion, decreased nasal airflow, epistaxis	Infiltrative mass that extends into nasopharynx	Cup forceps (15x10 mm)	COREAH (with AT)	None	Continued difficulty breathing	Euthanized (12)
7	11	Sneezing, epistaxis, stertor, facial swelling, ocular swelling	Extension of mass into nasopharynx, lysis of turbinates	Cup forceps (18x5 mm)	REAH	Asian ginseng, yarrow, burdock, dan shen gotu kola	Severe facial swelling persisted	Deceased (19.1)
8	6	Nasal congestion, ocular discharge	Body lysis, mass in nasal cavity, palate, and orbit; extension to contralateral side	Cup forceps (10 x5 mm)	COREAH	Aspirated viscous material from mass	Symptoms persisted, but patient stable	Alive (31)
9	5	Epistaxis, facial swelling, stertor, ocular discharge, decreased nostril airflow	Turbinate lysis, extension into frontal sinus	Rhinotomy (10x9 mm)	COREAH (with SH)	Saline rinse	Continued nasal congestion, ocular and nasal discharge	Deceased (18)
10	10	Sneezing, epistaxis, stertor, decreased nostril airflow	Extension through nasal cavity and into skull	Cup forceps (5x5 mm)	COREAH	Clavamox, enrofloxacin	Persistent sneezing, epistaxis, stertor	Euthanized (6.7)

11	7	Epistaxis, stertor, facial swelling, bilateral mandibular lymphadenopathy	Rhinosinusitis and multifocal turbinate lysis	Cup forceps (10x10 mm)	COREAH	Prednisone, Zyrtec	LTFU	LTFU
12	10	Epistaxis, nasal discharge, sneezing, difficulty breathing	Lysis of cribriform plate, orbit, and turbinates, extension into contralateral nasal cavity	Cup forceps (5x5 mm)	COREAH	Piroxicam	Continued difficulty breathing, nasal discharge	Deceased (3.2)
13	9	Epistaxis, decreased nasal airflow	Turbinate lysis, erosion of wall of orbit and cribriform plate, extension of mass into nasopharynx	Cup forceps (8x8mm)	COREAH (with AT)	Clindamycin	Persistent epistaxis	Alive (14)
14	3	Epistaxis, nasal discharge, difficulty breathing	Extension of mass to contralateral side, proliferation to cribriform plate	Rhinotomy (10x3 mm)	COREAH (with SH)	Antibiotics, steroids	Continued difficulty breathing and nasal discharge	Euthanized (2.7)
15	6	Congestion, mucopurulent discharge, decreased nasal airflow	Extension of mass into contralateral nasal passage	Cup forceps (10x10mm)	COREAH (with AT)	None	LTFU	LTFU
16	8	Sneezing, nasal discharge, stertor, congestion, epistaxis	Lysis of cribriform plate, deviation of nasal septum, extension of mass into contralateral nasal passage	Cup forceps (7x7 mm)	COREAH	CyberKnife	One episode of clear nasal discharge, otherwise no recurrence of symptoms	Alive (6)
17	7	Nasal discharge, increased respiratory effort, epistaxis	Turbinate lysis	Cup forceps (15x7 mm)	COREAH	Sinusotomy, debridement, Clavamox, carprofen, ablation procedure	No recurrence after ablation procedure	Alive (5)
18	13	Epistaxis	Soft tissue mass, suspected metastasis to medial retropharyngeal LN	Cup forceps (15x7 mm)	COREAH	Deramaxx	No recurrence of symptoms	Alive (5)

19	12	Upper respiratory signs, increased respiratory effort	Turbinate lysis, extension of mass into nasopharynx and contralateral nasal passage and sinus	Cup forceps (22x20 mm)	REAH	Chlorpheniramine, enrofloxacin, prednisone	Continued difficulty breathing	Alive (5)
20	9	Epistaxis, sneezing	Turbinate lysis, extension into contralateral nasal passage, globe displacement, mild mandibular lymphadenopathy	Cup forceps (23x6 mm)	COREAH	None	N/A	Euthanized (0 days)
21	8	Epistaxis, ocular discharge, decreased airflow from nostril	Soft tissue mass in left nasal cavity	Cup forceps (10x10 mm)	COREAH (with AT)	None	Not reported	Alive (2)

Abbreviations: CT, computed tomography; N/A, not applicable; LTFU, lost to follow-up; COREAH, chondro-osseous respiratory epithelial adenomatoid hamartoma; REAH, respiratory epithelial adenomatoid hamartoma; SH, seromucinous hyperplasia; AT, angiomatous tissue; LN, lymph node

[a]Histologic measurements of the largest samples evaluated.
